# Thickness, Annealing, and Surface Roughness Effect on Magnetic and Significant Properties of Co_40_Fe_40_B_10_Dy_10_ Thin Films

**DOI:** 10.3390/ma16175995

**Published:** 2023-08-31

**Authors:** Wen-Jen Liu, Yung-Huang Chang, Chia-Chin Chiang, Yuan-Tsung Chen, Yu-Zhi Wang, Chueh-Lin Wu, Shih-Hung Lin, Sin-Liang Ou

**Affiliations:** 1 Department of Materials Science and Engineering, I-Shou University, Kaohsiung 84001, Taiwan; jurgen@isu.edu.tw; 2 Bachelor Program in Industrial Technology, National Yunlin University of Science and Technology, 123 University Road, Section 3, Douliou 64002, Taiwan; changyhu@yuntech.edu.tw; 3 Department of Mechanical Engineering, National Kaohsiung University of Science and Technology, Kaohsiung 80778, Taiwan; ccchiang@nkust.edu.tw; 4 Graduate School of Materials Science, National Yunlin University of Science and Technology, 123 University Road, Section 3, Douliou 64002, Taiwan; u1042141@gmail.com (Y.-Z.W.); mr.wujay@gmail.com (C.-L.W.); 5 Department of Electronic Engineering, National Yunlin University of Science and Technology, 123 University Road, Section 3, Douliou 64002, Taiwan; isshokenmei@yuntech.edu.tw; 6 Bachelor Program for Design and Materials for Medical Equipment and Devices, Da-Yeh University, Changhua 51591, Taiwan; slo@mail.dyu.edu.tw

**Keywords:** Co_40_Fe_40_B_10_Dy_10_ thin films, annealing treatment, low frequency alternating current magnetic susceptibility (χ_ac_), optimal resonance frequency (f_res_), surface roughness, magnetic domain, surface energy, adhesion, transmittance

## Abstract

In this study, Co_40_Fe_40_B_10_Dy_10_ thin films were deposited using a direct current (DC) magnetron sputtering technique. The films were deposited on glass substrates with thicknesses of 10, 20, 30, 40, and 50 nm, and heat-treated in a vacuum annealing furnace at 100, 200, and 300 °C. Various instruments were used to examine and analyze the effects of roughness on the magnetic, adhesive, and mechanical properties. From the low frequency alternating current magnetic susceptibility (χ_ac_) results, the optimum resonance frequency is 50 Hz, and the maximum χ_ac_ value tends to increase with the increase in the thicknesses and annealing temperatures. The maximum χ_ac_ value is 0.18 at a film thickness of 50 nm and an annealing temperature of 300 °C. From the four-point probe, it is found that the resistivity and sheet resistance values decrease with the increase in film deposition thicknesses and higher annealing temperatures. From the magnetic force microscopy (MFM), the stripe-like magnetic domain distribution is more obvious with the increase in annealing temperature. According to the contact angle data, at the same annealing temperature, the contact angle decreases as the thickness increases due to changes in surface morphology. The maximal surface energy value at 300 °C is 34.71 mJ/mm^2^. The transmittance decreases with increasing film thickness, while the absorption intensity is inversely proportional to the transmittance, implying that the thickness effect suppresses the photon signal. Smoother roughness has less domain pinning, more carrier conductivity, and less light scattering, resulting in superior magnetic, electrical, adhesive, and optical performance.

## 1. Introduction

CoFe was discovered by Ellis in 1927 and Elmen in 1929; it has excellent properties of high saturation magnetization (Ms), low coercivity (Hc), and high magnetic permeability, and has been studied by many scientists [1]. CoFe alloy is a soft magnetic alloy with high hardness and brittleness in physical properties, and because it is easy to lose magnetic anisotropy after high temperature annealing treatment, this is its biggest disadvantage. In order to improve the various properties and resolve the issue, extra elements were added. The literature studies have pointed out that the addition of 3% of rare earths (La, Ce, Sm, Gd, Dy, Ho, Er, Yb) has an effect on the microstructure of the CoFe material, the phase content, and the magnetic performance [2,3]. In most studies, the magnetic characterization of CoFe alloys has received widespread attention because of its high saturation magnetization and higher Curie temperature (Tc). The addition of metalloid boron (B), as demonstrated in the literature, indicates that a large number of boron additives are conducive to the formation of an amorphous state. This also makes the film layer more dense and improves the corrosion resistance of the material [4]. By incorporating a modest amount of boron into CoFe alloys, soft ferromagnetic ternary alloys can be created. Some scientists have studied the application of ferromagnetic amorphous alloys (CoFeB) in the manufacture of magnetic recording heads and the magnetic field applied during the plating process in an attempt to adjust the magnetic anisotropy and permeability [5]. For example, low-temperature boron ions were added to CoFe_2_O_4_ spinel structures for fixing the magnetic field [6]. CoFeB is a soft magnetic alloy used to form a free or pinned layer in a magnetic tunnel junction (MTJ) with low coercivity, low damping coefficient, high saturation magnetization, and strong perpendicular magnetic anisotropy (PMA), which shows a very large tunneling magnetoresistance (TMR) because of its excellent properties and the addition of the fourth rare earth element dysprosium. This addition can either enhance the original properties or obtain different properties [7,8]. The limitations and durability of the components in which magnetic films are employed may be a crucial indicator of whether they are irrevocably harmed in high temperature conditions. Rare earth elements, which are ferromagnetic metals, frequently have distinct features that can be exploited to improve the thermal stability, and electrical properties of thin films at high temperatures. Their qualities can be utilized to improve thin film thermal stability, resistivity, and saturation magnetization at high temperatures. The rare earth element dysprosium (Dy) is added to CoFeB. The addition of Dy can increase the secondary phase and the lattice expansion leading to deformation, the Young’s coefficient is increased, the magnetostrictive strain is decreased, and the Curie temperature is increased which means that the thermal stability is increased [9]. Furthermore, Dy and B can be added to improve physical characteristics and magnetic exchange-coupling strength [10,11,12,13]. For these reasons, it is critical to investigate the addition of a third or fourth element. It is worthwhile to invest in the specific properties by incorporating Dy and B into CoFe alloys to generate CoFeBDy films. Surface roughness is a significant property for using the magnetic component at room temperature and annealing temperatures. It has a substantial impact on the physical properties of thin films, particularly ultrathin films. Researchers have investigated the role of roughness in magnetic, electrical, and optical properties [14,15,16,17]. The effect of surface roughness on magnetic, adhesive, and electrical properties is worth considering. In this study, a 10–50 nm thick Co_40_Fe_40_Dy_10_B_10_ film is deposited on a glass substrate and then annealed at 100, 200, and 300 °C. The main purpose of this work is to explore surface roughness in order to study the relationship between the surface energy and magnetic-optical properties of CoFeBDy thin films at varied thicknesses and annealing temperatures. The film structure, magnetic characteristics, surface energy, and optical properties following annealing treatment were finally investigated using the aforementioned preset parameters. Furthermore, the relevance of the examination is in investigating the surface roughness in relation to researching the surface energy and magnetic-optical features of CoFeBDy thin films at varied thicknesses and annealing temperatures.

## 2. Materials and Methods

Thin films of CoFeBDy with a thickness of 10–50 nm were sputtered onto a glass substrate by direct current (DC) magnetron sputtering at room temperature (RT). The films were prepared under the following four conditions: (a) room temperature (RT), (b) annealing at 100 °C for 1 h, (c) annealing at 200 °C for 1 h, and (d) annealing at 300 °C for 1 h. The power was 50 W and the power density was 1.65 W/cm^2^. The base pressure of the chamber was 3.54 × 10^−7^ Torr, and the working pressure of the Ar was 3.09 × 10^−3^ Torr. The air flow rate was 20 sccm and the loader speed was 20 rpm. Following deposition, the samples were annealed for 1 h at temperatures between 100 °C and 300 °C with a controlled heating rate of 30 °C/min and a cooling rate of 0.5 °C/min. The vacuum chamber pressure was held constant at 2.5 × 10^−3^ Torr during the annealing process. The target composition was 40at% Co, 40at% Fe, 10at% B, and 10at% Dy. A variety of sintered materials were used to create the test target. The CoFeBY target is a commercial alloy made from pure metals that was obtained from Gredmann Taiwan Ltd., Taipei City, Taiwan. The substrate and target were 30 cm apart. The target has a 2 mm thickness and a diameter of 3 inches. A powder combination was created using 99.9% pure elements of Co, Fe, B, and Y. The target composition ratio was approved by the original factory for composition testing. The Ar ion bombardment and the sputtering deposition ion angle were responsible for the discrepancy between the target’s composition and its actual composition [18]. In order to evaluate the structure of the CoFeBDy thin films, grazing incidence X-ray diffraction (GIXRD) patterns of Cuk1 (PAN analytical X’pert PRO MRD, Malvern Panalytical Ltd., Cambridge, United Kingdom) and low angle diffraction incidence of around 2° were used. The low frequency alternate current magnetic susceptibility (χ_ac_) instrument of Co_40_Fe_40_B_10_Dy_10_ thin films was investigated using a χ_ac_ analyzer (XacQuan). Standard samples were calibrated by external magnetic field χ_ac_ measurements. A χ_ac_ analyzer was used to test the samples. The range of the driving frequency was 10 to 25,000 Hz. Magnetization strength was used to calculate χ_ac_. For the purpose of preventing demagnetization, all samples were the same size and form. The χ_ac_ values are in arbitrary units (a.u.), since the exchange results are comparative values with respect to the reference standard samples. The relationship between magnetization and frequency was measured by χ_ac_ measurements. The χ_ac_ analyzer detects the best resonance frequency (f_res_) and reveals the frequency of the largest χ_ac_. The resistivity and sheet resistance (Rs) values were assessed using the conventional four-point method for electrical characteristics. The morphology of the films was examined using an atomic force microscope (AFM, NanoMagnetics Instruments, Ankara, Turkey, ezAFM), and the magnetic domains of the films were characterized using magnetic force microscopy (MFM). Three scanning repetitions at RT were used to evaluate AFM in non-contact mode with average area evaluation. The roughness value utilized is Ra. Ra represents the arithmetic mean deviation used to evaluate the area. The size of the scanning was 20 μm × 20 μm. Additionally, AFM was used to calibrate the precise thickness using the height difference method. Deionized (DI) water and glycerol were used to calculate the average contact angle (CAM-110; Creating Nano Technologies, Tainan City, Taiwan) by measuring three times. The contact angle was determined after the sample was taken out. Finally, the surface energy was computed using the contact angle [19,20,21]. A visible light source with a wavelength range of 500–800 nm was used with a Spectro Smart Analyzer to evaluate the optical properties.

## 3. Results

### 3.1. XRD Structure Property

**Figure 1:**
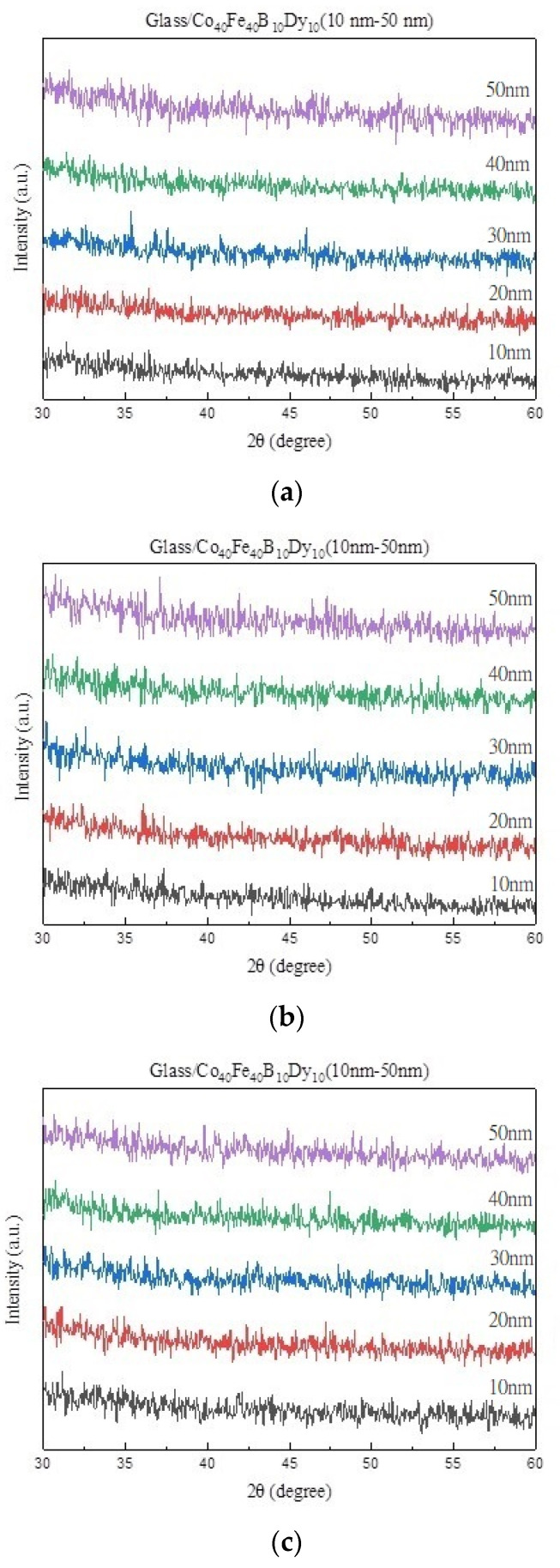

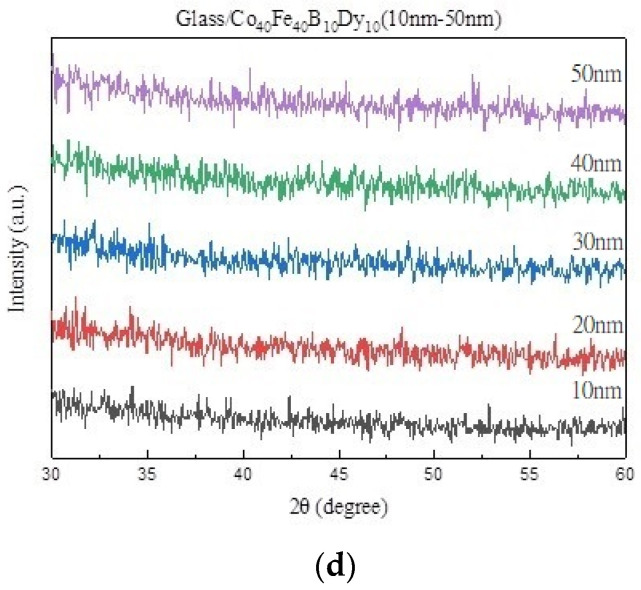
XRD patterns of Co_40_Fe_40_B_10_Dy_10_ films. (**a**) RT, (**b**) after annealing at 100 °C, (**c**) after annealing at 200 °C, (**d**) after annealing at 300 °C.

Figure 1a–d show the Co_40_Fe_40_B_10_Dy_10_ films with various thicknesses and annealing temperatures, and there are no significant peaks in the graphs, which means that the deposition on the glass substrate does not have any crystalline phases. According to the literature, it is proved that the addition of Dy and B to CoFe alloys causes the refinement of grain size when forming an amorphous structure [22,23]. Another reasonable reason is that the driving force of the heat treatment is insufficient to sustain grain growth to form crystals or the effect of the glass substrate itself is amorphous [24,25].

**Figure 2:**
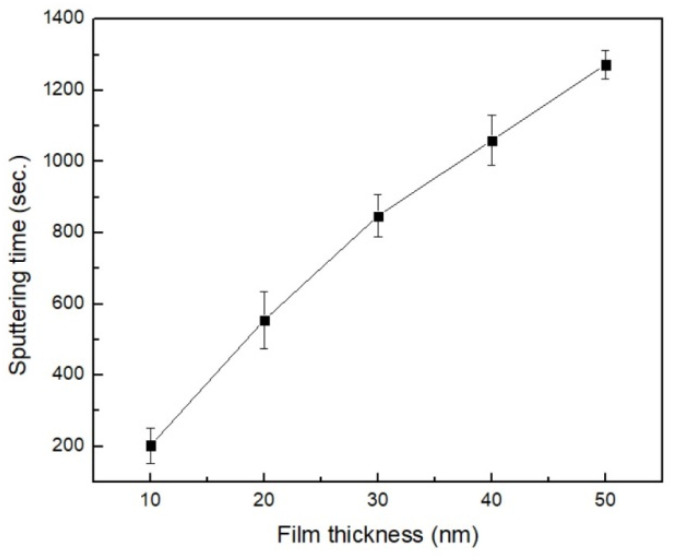
The calibrated thickness of sputtered time.

Figure 2 examines the calibrated thickness of the matching sputtering time in order to calibrate the accurate thicknesses. The diagram’s linear relationship implies that longer sputtering durations result in thicker films.

### 3.2. Magnetic and Electrical Properties

**Figure 3:**
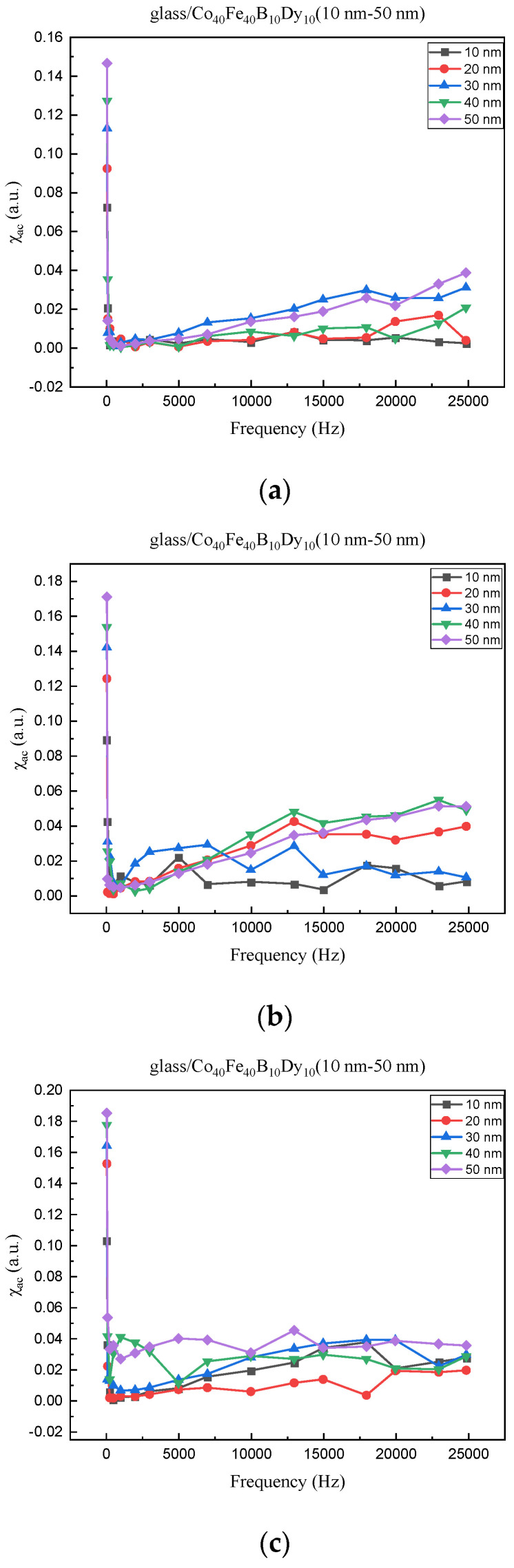

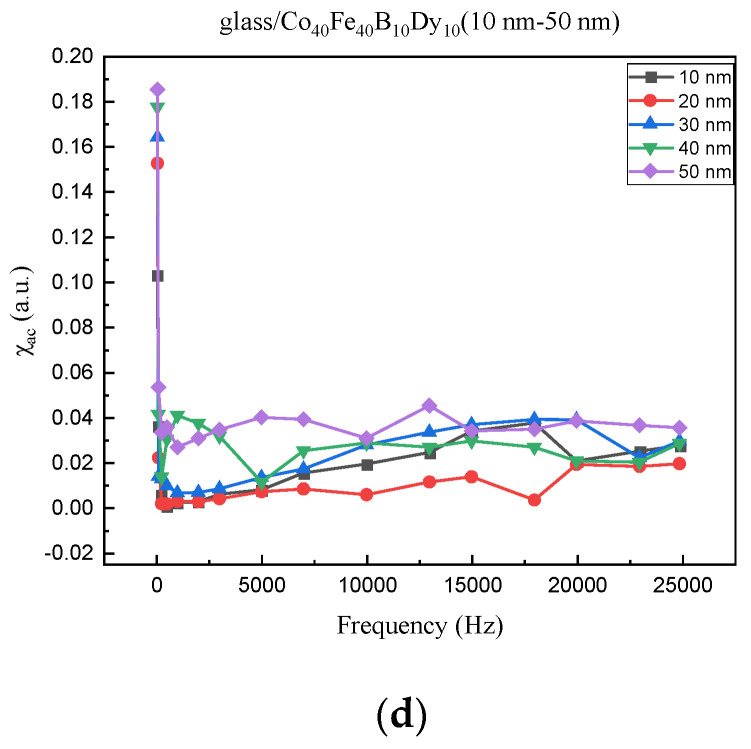
Low frequency alternate current magnetic susceptibility (χ_ac_) of Co_40_Fe_40_B_10_Dy_10_ films. (**a**) RT, (**b**) after annealing at 100 °C, (**c**) after annealing at 200 °C, (**d**) after annealing at 300 °C.

Figure 3a–d show the low frequency alternate current magnetic susceptibility (χ_ac_) values of Co_40_Fe_40_B_10_Dy_10_ films under four conditions. The χ_ac_ dropped as the frequency increased between 50 and 25,000 Hz. The matching χ_ac_ value increased as the thickness increased from 10 to 50 nm. It can be seen from the graphs that the maximum χ_ac_ values with or without heat treatment have an increasing trend due to the effect of the thickness [26]. The maximum χ_ac_ values are displayed in Table 1. The maximum χ_ac_ value at RT is 0.14 with a thickness of 50 nm, 0.15 at 100 °C with a thickness of 50 nm, 0.17 at 200 °C with a thickness of 50 nm, and 0.18 at 300 °C with a thickness of 50 nm. From the graphs, the maximum χ_ac_ values measured on the glass substrate are optimal at a resonance frequency of 50 Hz, but the χ_ac_ values tend to decrease with the increase in the measurement frequency, which is probably due to the anisotropy of the magnetic crystals [27,28]. At each thickness, the f_res_ value was 50 Hz. The optimal resonance frequency was determined to be less than 500 Hz, making it appropriate for usage in low frequency sensors, transformers, and magnetic components.

**Figure 4:**
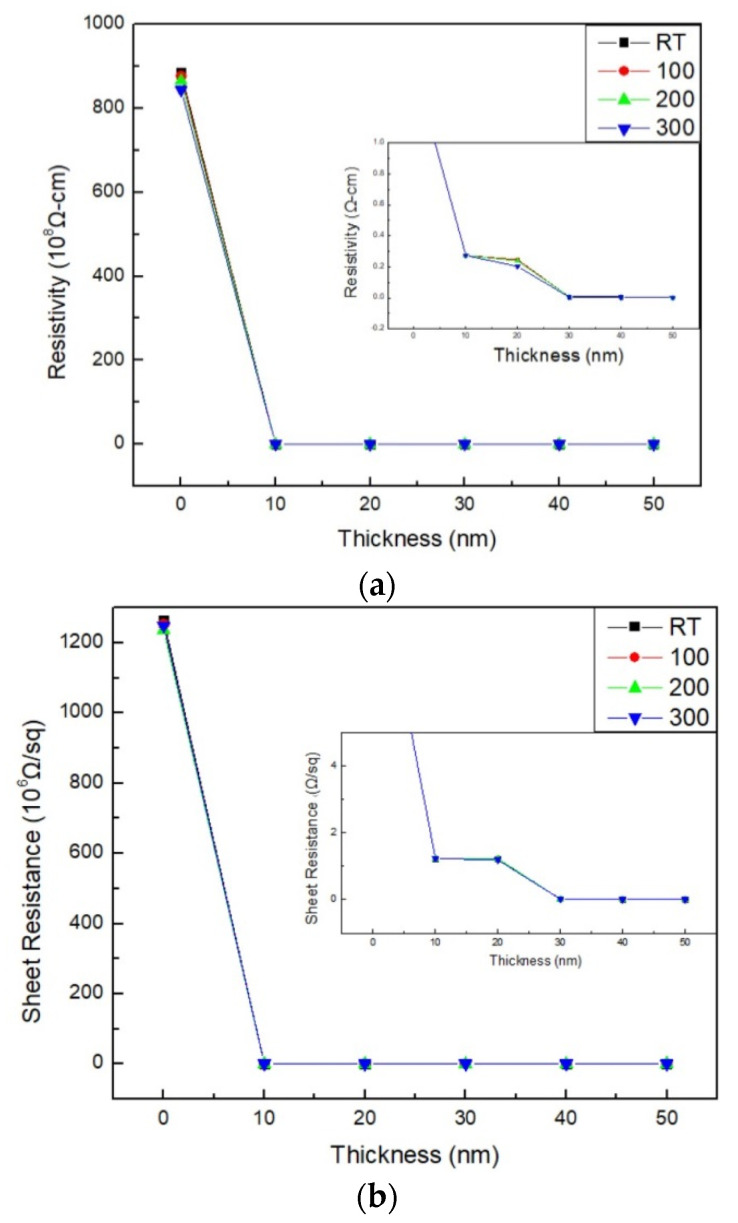
(**a**) Resistivity and (**b**) sheet resistance of Co_40_Fe_40_B_10_Dy_10_ films.

Figure 4a,b show the resistivity and sheet resistance. These have a significant downward trend with increasing thicknesses and annealing temperatures. The as-deposited and annealed conditions significantly impact the electrical characteristics of CoFeBDy films. Although higher annealing temperatures and thicker films appear to result in lower carrier hindrance, this causes lower resistance and is the main determinant of conductivity. According to the results of Figure 4, the resistivity ranged from 0.004 to 0.275 Ω-cm, while the sheet resistance ranged from 0.029 to 12.36 × 10^4^ Ω/sq. From the graph, the resistivity or sheet resistance has a constant value when the glass substrate is deposited at 10 nm or 20 nm. It is speculated that this is due to the thinner film thickness so that the current directly penetrates the film and passes through the substrate to obtain a constant value. As the thicknesses and annealing temperatures increase, the resistivity and sheet resistance decrease, which may be due to the greater thicknesses and annealing temperatures deposited so that the roughness decreases, which is shown in Table 2. The smooth surface roughness causes less hindrance of current carriers as they flow, resulting in a drop in electrical values [29,30]. As shown in Figure 4, the electrical resistivity and sheet resistance of glass substrate are substantially higher than the amount of deposited CoFeBDy on the glass substrate. It implied that the deposited CoFeBDy films were in a continuous state.

### 3.3. Surface Roughness and Magnetic Domain

This measurement is based on a thickness of 50 nm with the maximum χ_ac_ value, so the magnetic area distribution is the most obvious. It is pointed out that the more obvious tendency of the magnetic area distribution pattern is at greater thickness. Table 2 shows that the distribution of the magnetic area of Co_40_Fe_40_B_10_Dy_10_ is also in the shape of fine stripe, and the magnetic area is also more obvious with higher temperature treatment. The more obvious the stripe-like magnetic domain distribution, the smoother the surface roughness. On observing the 2D and 3D maps, it is found that the surface roughness is slightly smooth. The sample range for scanning is 20 μm × 20 μm. It is thought that the properties of the domain structures have a significant impact on the magnetic behaviors. With the film being annealed, the magnetic domain continuity and contrast are both enhanced. These are a result of the marginally improved exchange coupling [31,32]. Stronger magnetization is produced by the magnetic domain’s larger area, which is consistent with the χ_ac_ finding. In addition, the surface roughness Ra decreases from RT to 300 °C annealing. At RT, the Ra is 1.93 nm, while at 300 °C, it is 1.19 nm. From Figure 3 and results presented in Table 2, the maximum alternate current magnetic susceptibility and surface roughness have a relationship, indicating that increased surface roughness can cause the pinning effect of the domain wall, which makes it difficult to move and reduce the χ_ac_ value [33,34]. It is reasonable to conclude that the development of stripe magnetic domains results from the interaction of amorphous and nano-crystalline materials. The presence of an amorphous matrix with a higher density of nanograins will cause volume contraction, and the majority of amorphous substrates will thereby exert internal stresses on the grains, resulting in the development of magneto-elastic anisotropy and stripe domains [35,36].

### 3.4. Contact Angle and Surface Energy

**Figure 5:**
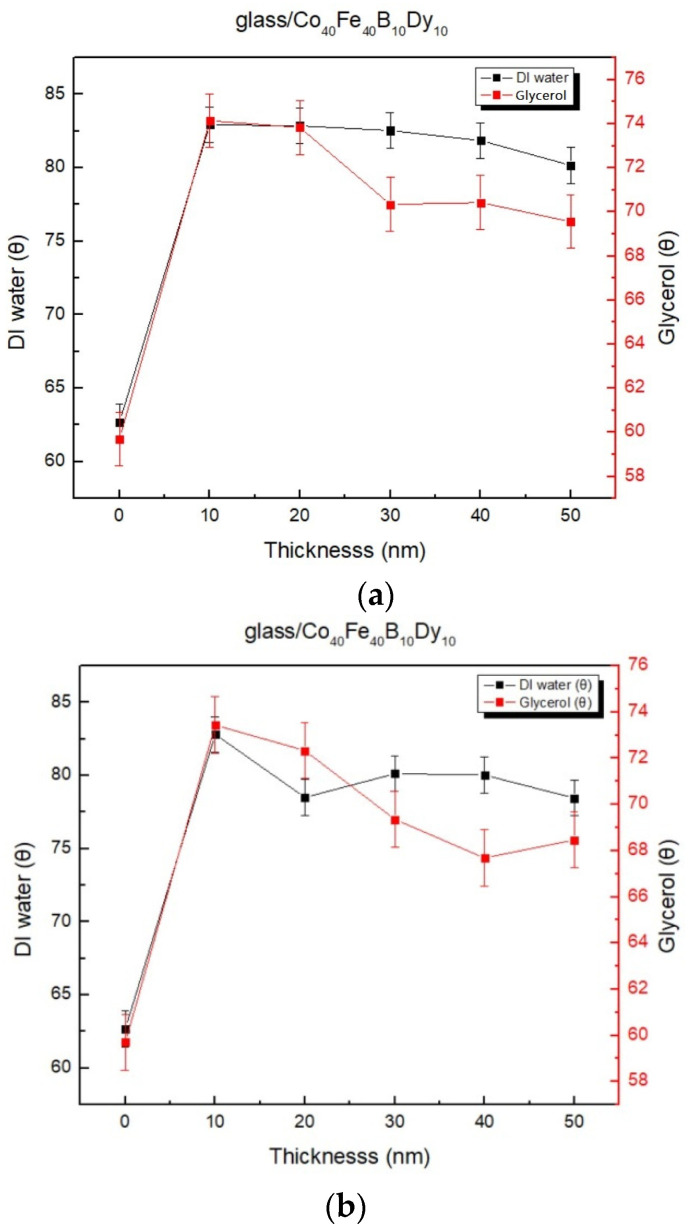

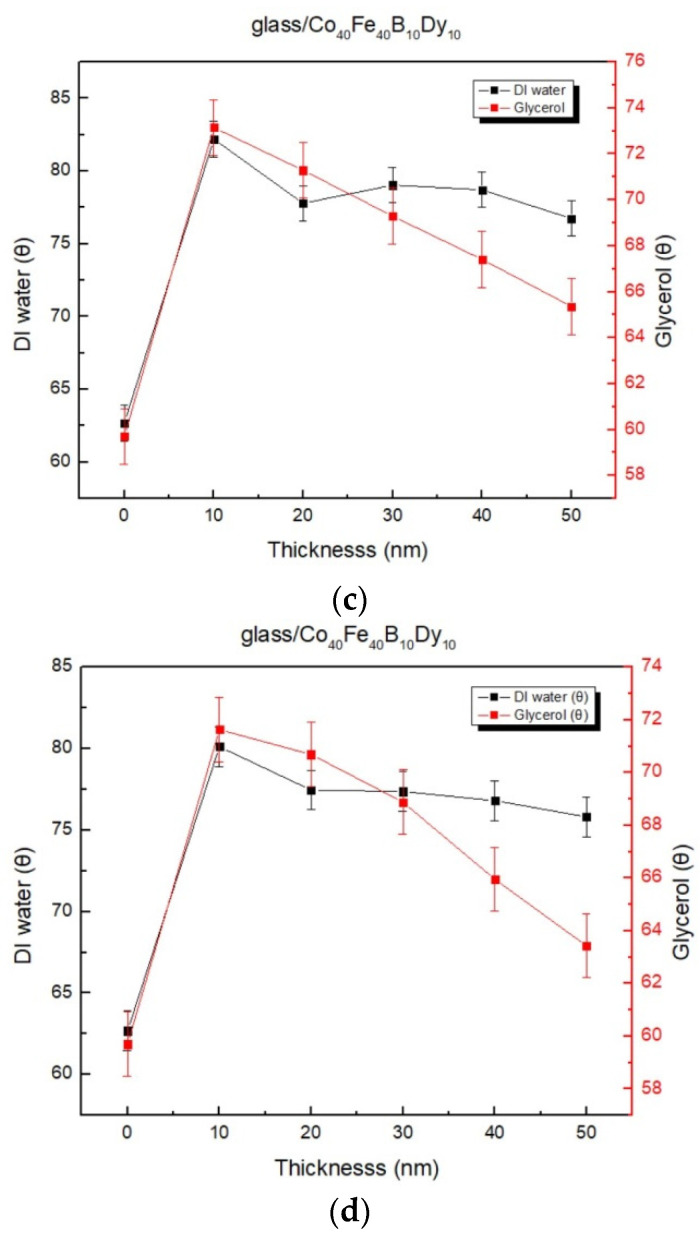
Contact angles of Co_40_Fe_40_B_10_Dy_10_ films. (**a**) RT, (**b**) after annealing at 100 °C, (**c**) after annealing at 200 °C, (**d**) after annealing at 300 °C.

The contact angle data from testing liquids such as DI water and glycerin are shown in Figure 5a through Figure 5d. The contact angles have a tendency to decrease when the annealing temperature is kept constant and the thickness of the film is increased. The contact angles also have a propensity to decrease at the same thickness and with higher annealing temperatures. This value is well-known in various solutions of Co_40_Fe_40_B_10_Dy_10_ film, where measured contact angles are less than 90°, which is proven to be a hydrophilic film and may have an impact on the surface contact angle of the two main components, including surface roughness and grain size of thin films. In this research, the higher annealing temperature results in decreased surface roughness and lower contact angle for the AFM at the same thickness [37,38,39].

**Figure 6:**
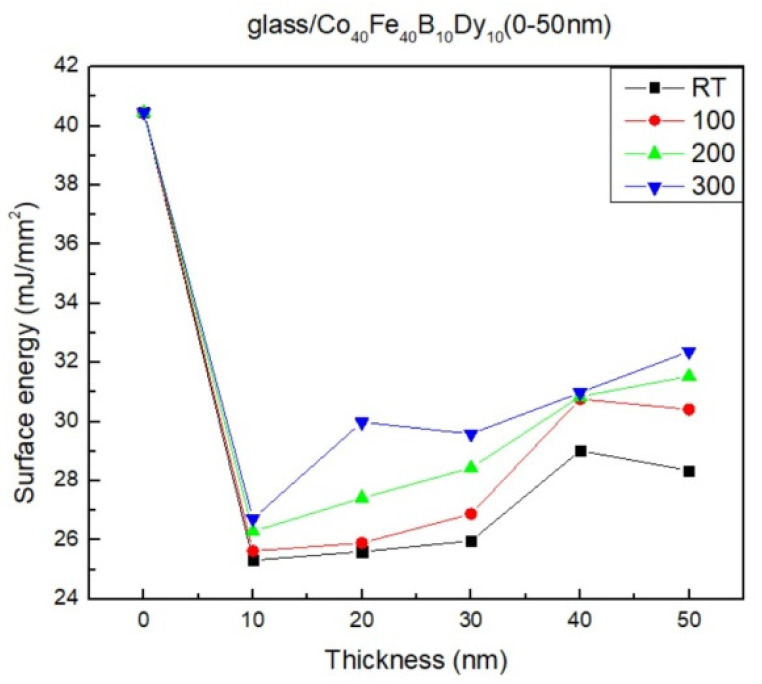
Surface energy of Co_40_Fe_40_B_10_Dy_10_ films.

Surface energy is depicted in Figure 6. The contact angle and Young’s equation are used to compute the surface energy [19,20,21]. Young’s equation is

σ_sg_ = σ_sl_ + σ_lg_ cosθ (1)

In the equation, σ_sg_ stands for the surface free energy of the solid, σ_sl_ stands for the interfacial tension between liquid and solid, σ_lg_ stands for surface tension of the liquid, and θ is for contact angle.

The surface energy increases with thickness and annealing temperature. According to the calculations, the greatest surface energy of 50 nm at 300 °C was 34.71 mJ/mm^2^. When the films had a higher surface energy, the adhesion was at its maximum. Due to the versatility of Co_40_Fe_40_B_10_Dy_10_ films as an underlayer or buffer layer, surface energy and adhesion are essential. The contact angle decreases, and liquid absorption is strong when the surface energy is high. The surface tension of the liquid decreases with increasing surface energy, and there is a stronger attraction between the molecules of the liquid and the atoms of the solid than between the molecules of the liquid themselves. Consequently, simpler wetting and greater adhesion result from higher surface energy [40]. As a result, the 50 nm Co_40_Fe_40_B_10_Dy_10_ film that had been annealed at 300 °C had greater adhesive qualities than the others, which might theoretically be explained by changes in surface morphology [41].

### 3.5. Optical Properties

**Figure 7:**
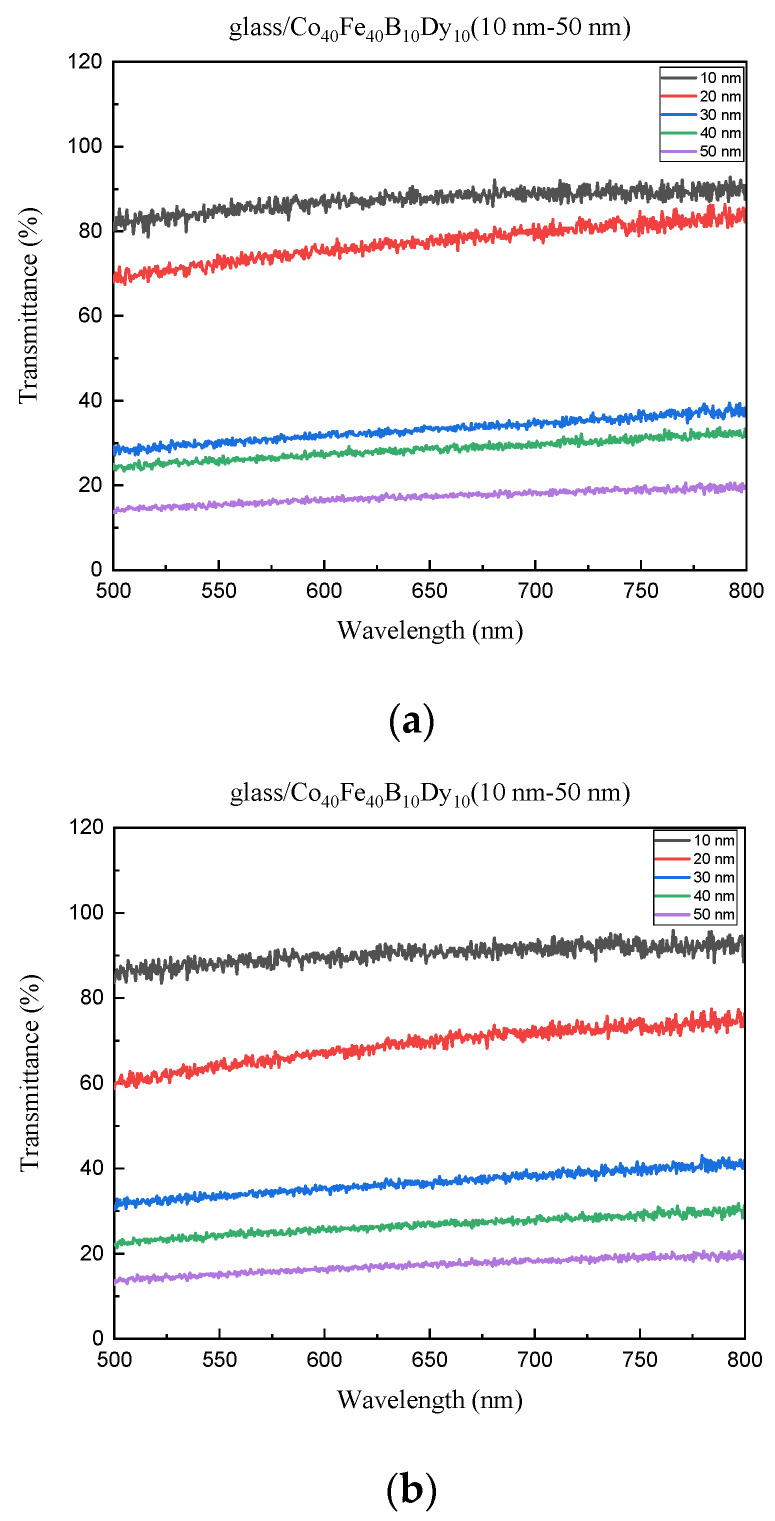

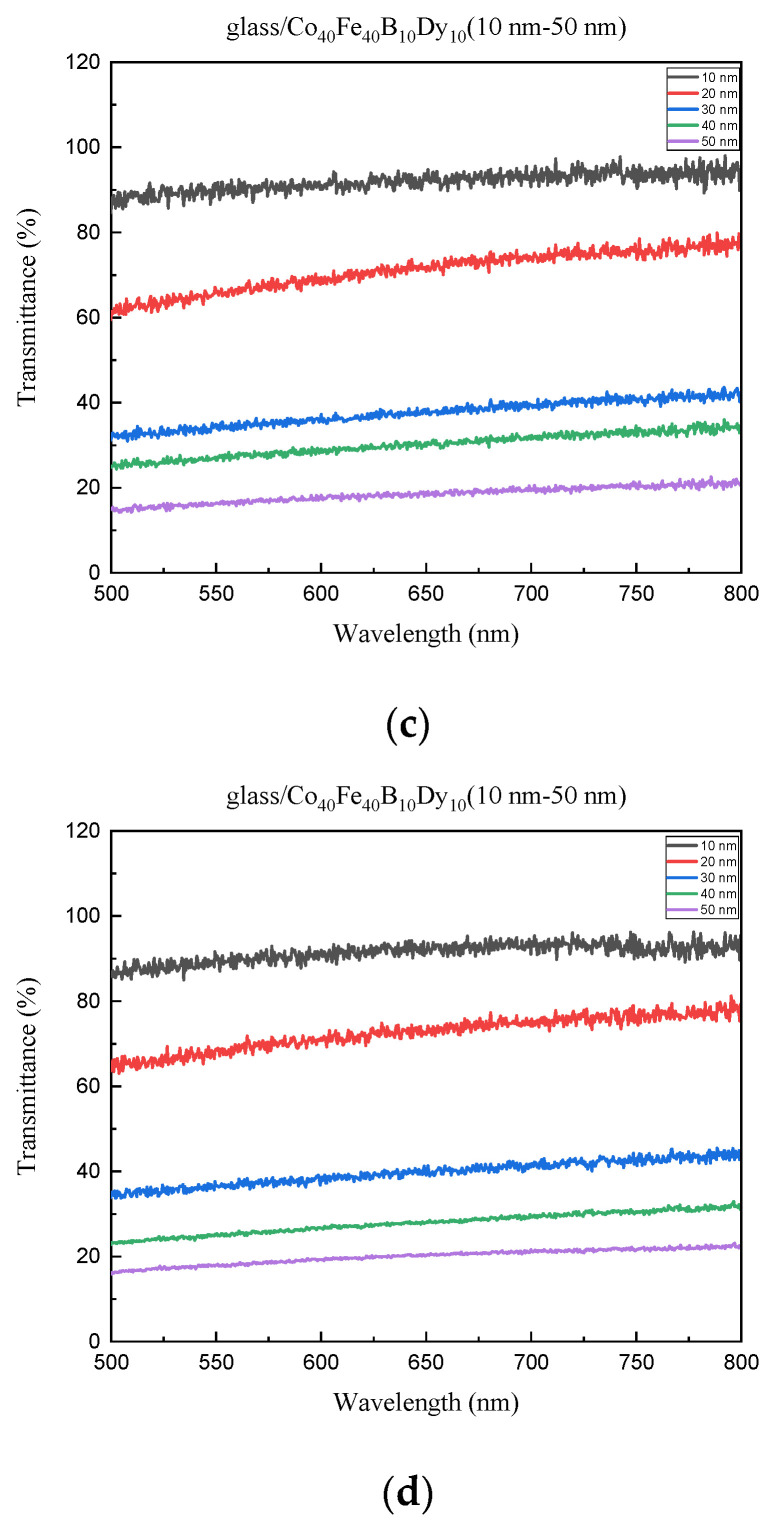
Transmittance of Co_40_Fe_40_B_10_Dy_10_ films. (**a**) RT, (**b**) after annealing at 100 °C, (**c**) after annealing at 200 °C, (**d**) after annealing at 300 °C.

As shown in Figure 7a, the transmittance of the film at RT declines from 81.83% to 13.49% as the thickness of the film increases. Figure 7b shows that when the film is annealed at 100 °C, the transmittance reduces from 84.59% to 13.40% as the thickness of the film increases. Figure 7c shows that when the film is annealed at 200 °C, the transmittance reduces from 86.71% to 14.71% as the thickness of the film increases. Figure 7d shows that for the film at 300 °C heat treatment, the transmittance falls from 86.03% to 15.63% as the thickness of the film increases. The transmittance decreased with increasing thickness from 10 nm to 50 nm, implying that a greater thickness may limit light signal passage through the films, resulting in high transmittance and low absorbance [42].

**Figure 8:**
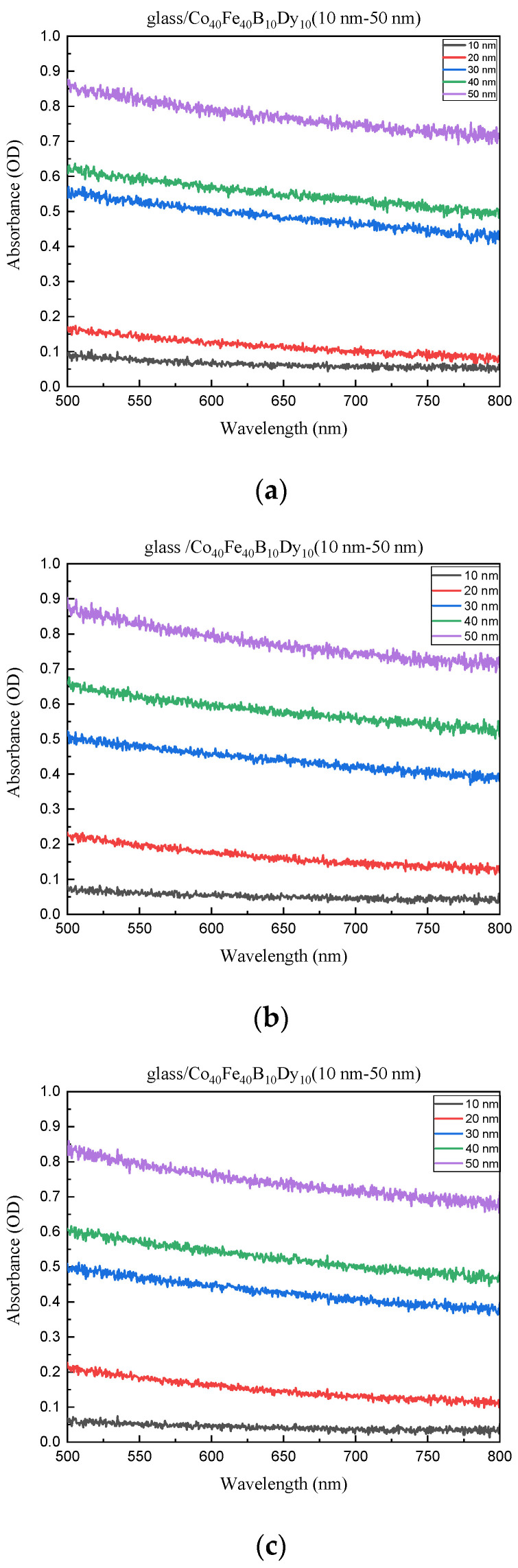

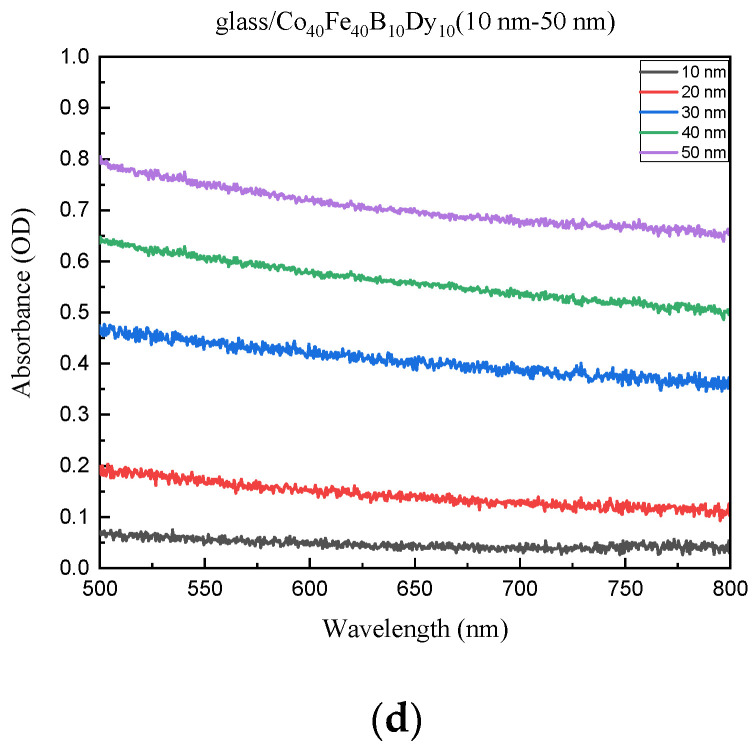
Absorbance of Co_40_Fe_40_B_10_Dy_10_ films. (**a**) RT, (**b**) after annealing at 100 °C, (**c**) after annealing at 200 °C, (**d**) after annealing at 300 °C.

The absorbance is depicted in Figure 8a through 8d under various conditions. Due to the effects of light dispersion caused by surface roughness, interference bands in optical absorption are not generally visible in thicker samples [43]. Greater light absorption and enhanced optical performance are implied by a smoother surface, which is compatible with the ZnO optical result [44]. Figure 8 shows that a greater thickness has a higher absorption intensity. The data demonstrate that the film’s transmittance is inversely related to its absorbance, and it is also clear from the literature that the thickness effect and the interface effect will suppress the light signal, which will have an impact on the penetration rate and reduce the transmittance [45,46,47]. In summary, the magnetic, electric, adhesive, and optical properties of CoFeBDy films depend significantly on their surface roughness at different annealing temperatures. The smoother roughness has less pinning effect on the domain wall, which makes it easier to move and enhance the χ_ac_ value. A lower contact angle and a greater surface energy are produced by the decreased surface roughness. Additionally, lessening light scattering and increasing carrier conductivity are two additional benefits of smoother roughness, which lead to higher transmittance and lower electrical resistivity. Furthermore, the effects of increasing thickness and annealing temperature also affect magnetic and optical properties. The thickness effect and annealing process both contribute to improving magnetic performance. Low transmittance and high absorbance are the effects of thickness and contact on light signal suppression.

## 4. Conclusions

Due to the presence of Dy and B as well as a lack of thermal driving force to enable grain formation, XRD of the Co_40_Fe_40_B_10_Dy_10_ films reveals that they have an amorphous structure. At all conditions, the best resonance frequency is 50 Hz. The χ_ac_ value tends to decrease with the increase in the measured frequency. The maximum χ_ac_ value tends to increase with the increase in thickness and annealing temperatures. The four-point probe determines that the film’s resistivity and sheet resistance values tend to decrease as the film deposition thickness and annealing temperature increase. AFM detection of a 50 nm Co_40_Fe_40_B_10_Dy_10_ film reveals a stripe-like magnetic domain distribution as the heat treatment temperature rises. The surface roughness Ra value decreases with increasing annealing temperatures at 50 nm. The contact angle measurements revealed that all the Co_40_Fe_40_B_10_Dy_10_ films exhibited hydrophilic properties. At 300 °C and 50 nm, the maximum surface energy is attained. Because of the thickness effect and the interface effect, the transmittance of the film decreases with thickness and marginally increases from short wavelength to long wavelength. Smoother roughness has less domain pinning, more carrier conductivity, and less light scattering, resulting in superior magnetic, electrical, adhesive, and optical performance. In this investigation, the best condition is 50 nm annealed at 300 °C. The incorporation of dysprosium (Dy) into a CoFe has been shown to enhance thermal stability. Therefore, it is recommended that researchers investigate the thermal stability of the fabricated thin film, as this property holds considerable significance in the context of the study.

## Figures and Tables

**Table 1:** Maximum χ_ac_ values of Glass/Co_40_Fe_40_B_10_Dy_10_ (10~50 nm) thin films at different temperatures.

	Temperature	RT Maximum (a.u.)	100 °C Maximum (a.u.)	200 °C Maximum (a.u.)	300 °C Maximum (a.u.)
Thickness					
10 nm		0.07	0.08	0.08	0.10
20 nm		0.09	0.10	0.12	0.15
30 nm		0.11	0.12	0.14	0.16
40 nm		0.12	0.13	0.15	0.17
50 nm		0.14	0.15	0.17	0.18

**Table 2:** Co_40_Fe_40_B_10_Dy_10_ 50 nm films at different annealing temperatures with MFM images and surface roughness.

Temperature (°C)	Magnetic Domain	Surface Roughness (nm)	Average Roughness, Ra (nm)
RT	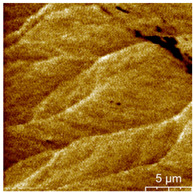	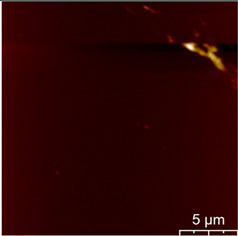 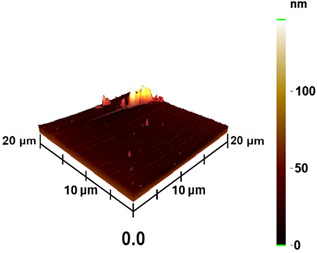	1.93 nm
100	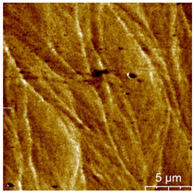	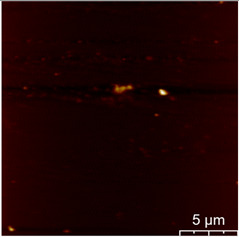 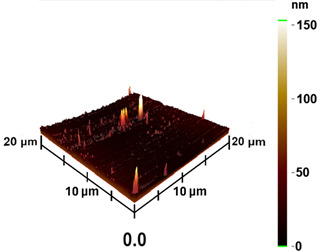	1.79 nm
200	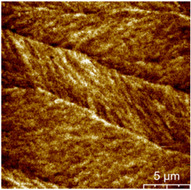	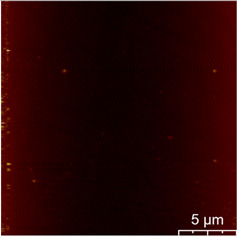 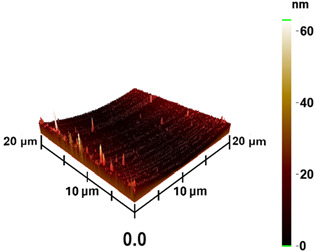	1.71 nm
300	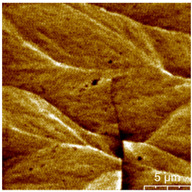	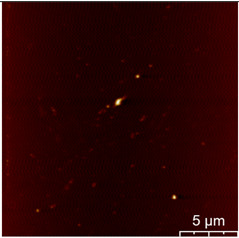 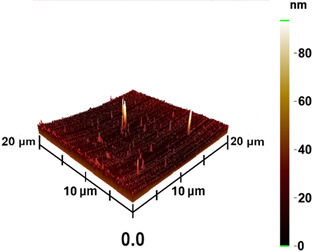	1.19 nm

## Data Availability

Not applicable.
